# Recent Advances in Antibacterial Strategies Based on TiO_2_ Biomimetic Micro/Nano-Structured Surfaces Fabricated Using the Hydrothermal Method

**DOI:** 10.3390/biomimetics9110656

**Published:** 2024-10-26

**Authors:** Zilin Guo, Hanpeng Liu, Wuzhi Wang, Zijun Hu, Xiaofang Li, Hao Chen, Kefeng Wang, Zhaoyang Li, Caideng Yuan, Xiang Ge

**Affiliations:** 1 Key Laboratory of Mechanism Theory and Equipment Design of Ministry of Education, School of Mechanical Engineering, Tianjin University, Tianjin 300354, China; 2 Tianjin Key Laboratory of Composite and Functional Materials, School of Materials Science and Engineering, Tianjin University, Tianjin 300072, China; 3 College of Foreign Languages, Taiyuan University of Technology, Taiyuan 030024, China; 4 State Key Laboratory of Mechanical System and Vibration, School of Mechanical Engineering, Shanghai Jiao Tong University, Shanghai 200240, China; 5 National Engineering Research Center for Biomaterials, Sichuan University, Chengdu 610064, China; 6 School of Chemical Engineering and Technology, Tianjin University, Tianjin 300350, China

**Keywords:** antibacterial strategies, titanium dioxide, hydrothermal method, biofabrication, orthopedic, implants

## Abstract

Ti and its alloys, widely utilized in orthopedic and dental implants, inherently lack antibacterial properties, posing significant infection risks, especially in the context of growing antibiotic resistance. This review critically evaluates non-antibiotic antibacterial strategies, with a particular focus on surface modifications and micro/nano-structured surfaces. Micro/nano-structured surfaces, inspired by natural topographies, utilize physical mechanisms to eradicate bacteria. Despite their potential, the antibacterial efficacy of these surfaces remains insufficient for clinical application. Titanium dioxide (TiO_2_), known for its excellent photocatalytic antibacterial activity and biocompatibility, is emerging as an ideal candidate for enhancing micro/nano-structured surfaces. By combining the photocatalytic antibacterial effects of TiO_2_ with the mechanical bactericidal properties of micro/nano-structured surfaces, superior antibacterial performance can be achieved. The hydrothermal method is frequently employed to fabricate TiO_2_ micro/nano-structured surfaces, and this area of research continues to thrive, particularly in the development of antibacterial strategies. With demonstrated efficacy, combined antibacterial strategies based on TiO_2_ micro/nano-structured surfaces have become a prominent focus in current research. Consequently, the integration of physical stimulation and chemical release mechanisms may represent the future direction for TiO_2_ micro/nano-structured surfaces. This review aims to advance the study of TiO_2_ micro/nano-structured surfaces in antibacterial applications and to inspire more effective non-antibiotic antibacterial solutions.

## 1. Introduction

In recent decades, the growing demand for orthopedic and dental implants reflects the global population’s aging and the improvement in medical standards. Projections indicate that by 2030, the United States will experience a dramatic increase in primary total knee replacements, estimated at 3.48 million, representing a 673% rise from 2005 levels [1]. Titanium (Ti) and its alloys are prominent biomaterial choices for these implants, possessing characteristics such as low Young’s modulus, extended fatigue life, strong corrosion resistance, exceptional mechanical strength, and commendable biocompatibility [2,3]. However, a significant drawback of Ti and its alloys is their lack of inherent antibacterial properties, which poses challenges in clinical applications [4].

Implant-associated infections (IAIs) are a leading cause of implant failure, posing substantial risks to patients [5,6]. Statistics from surgical care improvement projects highlight the ongoing challenge of reducing infection rates in elective surgeries to below 1–2% [7]. The risk escalates when patients develop infections that require revision surgery, exacerbating psychological, physiological, and financial burdens threefold [8]. IAIs place significant strain on both patients and medical institutions, prompting the medical community to confront the formidable task of effective prevention and treatment [9,10]. 

**Figure 1:**
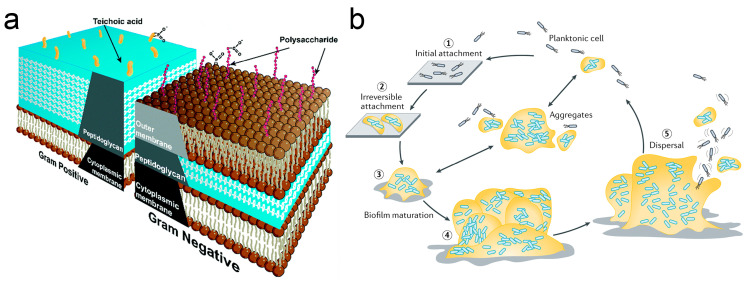
(**a**) Schematic diagram illustrating the cell wall structures of Gram-positive and Gram-negative bacteria [13]; and (**b**) The biofilm formation process based on single-species *P. aeruginosa* [15]. The attachment process of planktonic bacteria to surfaces primarily involves five stages: initial attachment (Step ①), irreversible attachment (Step ②), biofilm maturation (Steps ③ and ④), and dispersion (Step ⑤). In Step ①, bacteria initially attach to surfaces at their cell poles or via flagella. In Step ②, the attachment becomes irreversible as bacteria anchor themselves to the surface and begin producing biofilm matrix components. Step ③ marks the gradual maturation of cell clusters formed by multiple bacteria. In Step ④, the biofilm reaches full maturity. Finally, in Step ⑤, the biofilm disperses, allowing bacteria to enter a new biofilm formation cycle. Reproduced with permission from Refs. [13,15].

The primary bacterial pathogens responsible for IAIs include Gram-positive bacteria, such as *Staphylococcus aureus* (*S. aureus*), and Gram-negative bacteria, represented by *Escherichia coli* (*E. coli*) and *Pseudomonas aeruginosa* (*P. aeruginosa*) [11,12]. Gram-positive and Gram-negative bacteria exhibit distinct cell wall compositions [13], as illustrated in Figure 1a. Generally, Gram-negative bacteria have a thinner peptidoglycan layer compared to Gram-positive bacteria, complemented by an additional hydrophobic lipid bilayer [13]. The structure of the bacterial cell wall plays a crucial role in determining the interaction between bacteria and the surface of implants [13].

Bacterial infections on implant surfaces typically manifest in the form of bacterial biofilms [14]. Planktonic bacteria adhere to the implant surface and undergo processes of aggregation and proliferation, ultimately forming biofilms [12,15], as illustrated in Figure 1b. Biofilms represent robust bacterial communities that express additional virulence factors, making their removal significantly more challenging than that of planktonic bacteria [12]. Consequently, biofilms constitute a substantial threat to implants [12,15].

Currently, oral or intravenous antibiotics are the primary methods for reducing IAIs [16]. However, delivering adequate antibiotic concentrations to the target surfaces of implants presents significant challenges [16]. The formation of biofilms diminishes the efficacy of conventional systemic or topical antibiotic treatments, as disrupting biofilms requires substantially higher antibiotic doses than those needed to eliminate single-celled planktonic bacteria [12,15,17]. In many cases, antibiotics alone are insufficient for biofilm eradication [12,18]. Furthermore, the overuse and misuse of antibiotics have contributed to the rise of bacterial resistance, exemplified by the emergence of methicillin-resistant *S. aureus* (MRSA) and multidrug-resistant “superbugs” [19]. Antibiotic resistance poses a global threat, compromising the effectiveness of these medications [19]. Consequently, there is an urgent need to explore non-antibiotic antibacterial strategies that enhance the antibacterial properties of implants while preserving their normal functionalities, thereby further mitigating the risk of IAIs.

**Figure 2:**
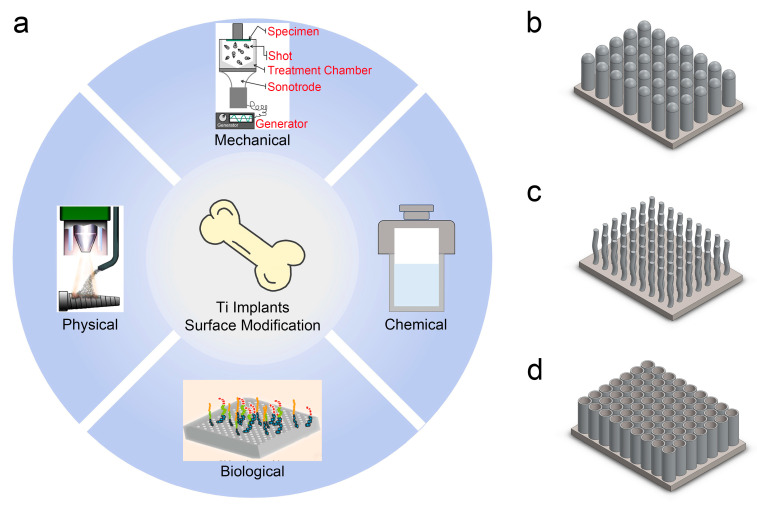
(**a**) Surface modification techniques for Ti implants: mechanical, physical, chemical, and biological treatments [25,26,28]; (**b**) Nano-pillar array model; (**c**) Nano-wire array model; and (**d**) Nanotube array model. Reproduced with permission from Refs. [25,26,28].

Surface modification of Ti implants is an effective strategy to eradicate bacteria and inhibit biofilm formation [20], as illustrated in Figure 2a. Based on the underlying mechanisms, surface modification techniques can be broadly categorized into mechanical, physical, chemical, and biological methods [21,22,23,24]. Mechanical methods, such as surface grinding and polishing, involve the mechanical processing of the Ti surface. Novel techniques like surface mechanical attrition treatment also fall under this category [21,25]. Physical methods modify the Ti surface structure through processes like laser treatment and plasma spraying, enhancing surface properties without altering the chemical characteristics [22,26]. Chemical methods involve altering the surface composition through reactions, such as those employed in electrochemical [27] and hydrothermal treatments [23]. Finally, biological methods, commonly used in biomedicine, are employed to immobilize or infiltrate drugs or biological materials on the surface [24,28]. A wide variety of Ti implant surface coatings and structures have been developed through these surface-modification techniques [29]. The combined use of two or more surface modification techniques is a potential strategy to eradicate bacteria [30]. Microrough implants are widely used in clinical practice, and numerous in vitro and in vivo studies have confirmed that nanoscale modifications of Ti implants enhance bioactivity [31,32].

In recent years, the antibacterial efficacy of biomimetic micro/nano-structured surfaces has attracted significant attention from researchers. Following Ivanova et al.’s discovery that micro/nano-structures on cicada wings can exterminate *P*. *aeruginosa* [33], the bactericidal potential of natural biological surfaces, such as dragonfly wings and gecko skin, has been extensively studied [34,35,36,37]. Inspired by these natural surfaces, researchers have developed a diverse array of biomimetic micro/nano-structured surfaces, as depicted in Figure 2b–d. Studies have shown that the physical and mechanical interactions between these biomimetic surfaces and bacteria not only eradicate bacteria but also inhibit bacterial adhesion and biofilm formation [38]. Micro/nano-structured antibacterial surfaces typically possess a larger specific surface area [3,14]. Furthermore, high aspect ratio micro/nano-structured surfaces can inhibit microorganism growth through physical and mechanical mechanisms [14]. In contrast to macroscopic surfaces, micro/nano-structured surfaces can impede the initial attachment of microorganisms and the formation of biofilms [14,31]. The micro- and nanoscale topographical features on implant surfaces are expected to significantly enhance antibacterial efficacy [39,40].

Achieving a broad-spectrum antibacterial surface with micro/nano-structures remains an ideal goal [14]. While the surface roughness of these structures influences bacterial adhesion and biofilm formation, its effects can vary among different bacterial species [41,42]. Additionally, high surface roughness may exert differing impacts on bacteria and cells [43]. Therefore, a key research challenge is to fabricate micro/nano-structured surfaces with broad antibacterial properties for Ti implants.

The photocatalytic antibacterial properties of titanium dioxide (TiO_2_), a natural oxide present on the surface of Ti, have been extensively investigated [44]. TiO_2_ is an n-type semiconductor known for its excellent photocatalytic activity and light stability, making it widely used in sterilization and disinfection [45]. Through photocatalytic action, TiO_2_ generates reactive oxygen species (ROSs) that disrupt bacterial cell membranes, demonstrating significant antibacterial effects against a wide range of bacteria [44]. Additionally, TiO_2_ offers advantages such as good biocompatibility and low toxicity, making it highly suitable for applications on Ti implants [46].

Inspired by these findings, researchers have sought to integrate biomimetic micro/nano-structured surfaces with the photocatalytic properties of TiO_2_ to develop a promising non-antibiotic antibacterial strategy for treating IAIs. This approach leverages the combined physical-mechanical bactericidal effects and biofilm inhibition capabilities of biomimetic micro/nano-structured surfaces, along with the excellent photocatalytic antibacterial properties of TiO_2_.

Studies have demonstrated that micro/nano-structured surfaces exhibit higher photocatalytic activity compared to nanoparticles (NPs), as photo-excited electrons possess enhanced reducing abilities [47]. Hierarchical nano-structures further increase photocatalytic activity due to greater light reflection and internal light scattering [48]. The application of TiO_2_ biomimetic micro/nano-structured surfaces on implants holds significant promise for combating IAIs.

Among the various biofabrication methods for TiO_2_ biomimetic micro/nano-structured surfaces, hydrothermal methods are notable for their simplicity, cost-effectiveness, low environmental impact, and high product purity [49]. This method enables the fabrication of TiO_2_ structures such as nano-pillars [50], nano-spikes [51,52], and nano-sheets [53] by adjusting experimental parameters. Notably, hydrothermal methods offer advantages in topography selection and precise control of parameters, making them highly effective for producing desired surface structures [50].

The aim of this review paper is to present the research advancements in TiO_2_ biomimetic micro/nano-structured surfaces prepared via the hydrothermal method and explore their potential and future prospects for enhancing the antibacterial properties of Ti implants. Several representative natural antibacterial surfaces and their underlying antibacterial principles have been introduced. Additionally, artificial antibacterial surfaces and their mechanical bactericidal effects have also been discussed. Despite ongoing exploration of the antibacterial potential of micro/nano-structured surfaces, their widespread antibacterial effects remain limited. The challenges faced by micro/nano-structured surfaces in achieving robust antibacterial effects have been addressed.

TiO_2_ exhibits exceptional photocatalytic antibacterial properties, influenced by its crystal structure and topography [54]. A detailed overview of the crystal structure and photocatalytic antibacterial mechanism of TiO_2_ has been provided. The antibacterial ability of TiO_2_ has been widely applied in the biomedical field. Furthermore, how combining TiO_2_ with biomimetic micro/nano-structured surfaces represents a promising strategy for reducing IAIs has been discussed.

For biofabrication methods, the hydrothermal method has been categorized into alkaline and acidic types, summarizing the characteristics and advantages of each for preparing TiO_2_ biomimetic micro/nano-structured surfaces. The research progress on TiO_2_ biomimetic micro/nano-structured surfaces prepared via the hydrothermal method has been systematically analyzed with a focus on enhancing antibacterial efficacy.

Current research primarily explores combined antibacterial strategies that leverage both chemical and photo-excited antibacterial activities. However, the full potential of TiO_2_ biomimetic micro/nano-structured surfaces for implants remains to be fully explored. Lastly, the application value and prospects of TiO_2_ biomimetic micro/nano-structured surfaces in biomedicine has been adequately discussed, highlighting their potential to address various medical challenges.

## 2. Antibacterial Efficacy of Biomimetic Micro/Nano-Structured Surfaces

The eradication of infectious diseases, particularly those caused by bacteria, has long been a formidable challenge. This difficulty is compounded by the formation of bacterial biofilms, which confer resistance to conventional antibacterial treatments. In the quest to combat bacterial infections, researchers have turned to nature for inspiration. Natural organisms have evolved intricate mechanisms to prevent bacterial adhesion and colonization on their surfaces. Examples include the antibacterial properties exhibited by the surfaces of cicada wings [33,55], dragonfly wings [34,36], gecko skin [35], lotus leaves [56], and shark skin [57]. The antibacterial efficacy of biomimetic micro/nano-structured surfaces has been extensively studied.

### 2.1. Typical Natural Antibacterial Surfaces

Through in-depth investigation of their underlying mechanisms, researchers have categorized natural antibacterial surfaces into two main types: bactericidal surfaces and antibacterial adhesion surfaces [58].

#### 2.1.1. Natural Bactericidal Surfaces

**Figure 3:**
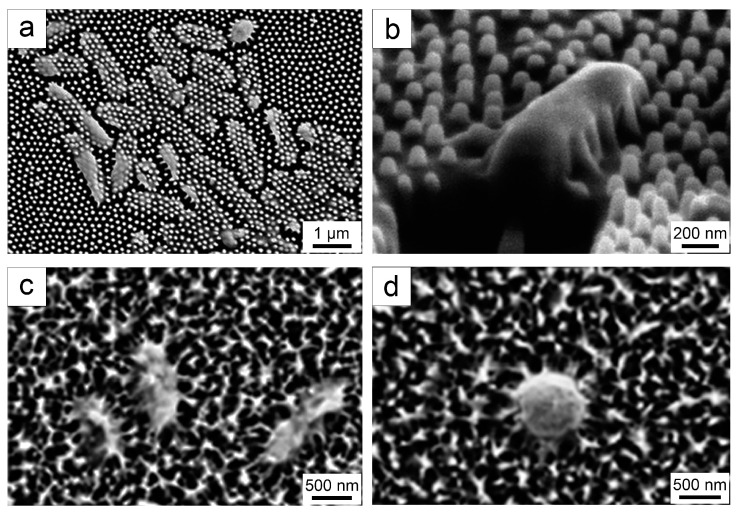
The antibacterial efficacy of typical natural bactericidal surfaces. (**a**) A top view shows that the bacteria (*P. aeruginosa*) have been punctured by the nano-pillars on the cicada wing surface [33]; (**b**) A cross-sectional view illustrates the interaction details between the bacteria (*P. aeruginosa*) and the nano-pillars, which was obtained by employing the focused ion beam scanning electron microscopy (FIB-SEM) [33]; (**c**) Bactericidal effect of dragonfly wing surfaces against *P. aeruginosa* [34]; and (**d**) Bactericidal effect of dragonfly wing surfaces against *S. aureus* [34]. Reproduced with permission from Refs. [33,34].

Bactericidal surfaces possess the capability to directly exterminate bacteria adhering to the surface [58]. This bactericidal ability is typically achieved through either physical or chemical means [58]. Here, we focus primarily on biological surfaces that employ mechanical bactericidal ability. Mechanical bactericidal ability is characterized by the interaction between micro/nano-structured surfaces and bacteria [59]. Through penetration and stretching of bacterial cells, the nanoscale protrusions present on bactericidal surfaces disrupt the structural integrity of the bacteria, leading to their demise [60,61]. Figure 3 illustrates the antibacterial efficacy of typical natural bactericidal surfaces.

In 2012, the physical-mechanical bactericidal ability of the *Psaltoda claripennis* cicada wing surface has been first discovered [33]. The nano-pillar arrays on this surface have been found to effectively eliminate *P. aeruginosa* (Figure 3a,b) [33]. Moreover, it has been demonstrated that the bactericidal effect of this surface remains unaffected by the chemical composition when gold coating has been applied using magnetron sputtering [33]. The nano-pillar arrays comprise hexagonal spherical conical nano-pillars with an average height of approximately 200 nm, a bottom diameter of around 100 nm, and a top diameter of about 60 nm [33]. The spacing between the nano-pillars is approximately 170 nm [33]. Subsequent studies on various cicada wing surfaces have demonstrated their ability to eradicate *Pseudomonas fluorescens* (*P. fluorescens*) and the eukaryotic microorganism *Saccharomyces cerevisiae* (*S. cerevisiae*) [55,62,63].

The *Diplacodes bipunctata* dragonfly wing surface has been demonstrated to exhibit bactericidal ability against both the Gram-positive bacterium *S. aureus* and the Gram-negative bacterium *P. aeruginosa* (Figure 3c,d) [34]. The nano-protrusions present on this dragonfly wing surface form a network at the lower end and clusters at some of the tips, with a height of approximately 240 nm [37]. This bactericidal effect has also been observed on various other dragonfly wing surfaces [34]. The nano-protrusions on these surfaces measure about 200–300 nm in height, with a top diameter of about 80 nm and a spacing of around 180 nm [34]. Additionally, the *Orthetrum villosovittatum* dragonfly wing possesses layered nano-pillar structures and exhibits natural bactericidal ability against *E. coli* [36].

Unlike the *Psaltoda claripennis* cicada wing and *Diplacodes bipunctata* dragonfly wing surfaces, the gecko skin surface exhibits a hierarchical nano-structure differentiated by height [35]. The nano-spikes on the surface have a height of approximately 2 μm and demonstrate bactericidal ability against both Gram-positive and Gram-negative bacteria [35]. Furthermore, it has been demonstrated using acrylic replicas that the bactericidal ability of these nano-spikes is primarily attributed to the topographical effect of the nano-structures [35].

Throughout evolution, natural biological surfaces have developed diverse bactericidal properties. These surfaces primarily eliminate bacteria through physical-mechanical interactions with bacterial cells.

#### 2.1.2. Natural Antibacterial Adhesion Surfaces

**Figure 4:**
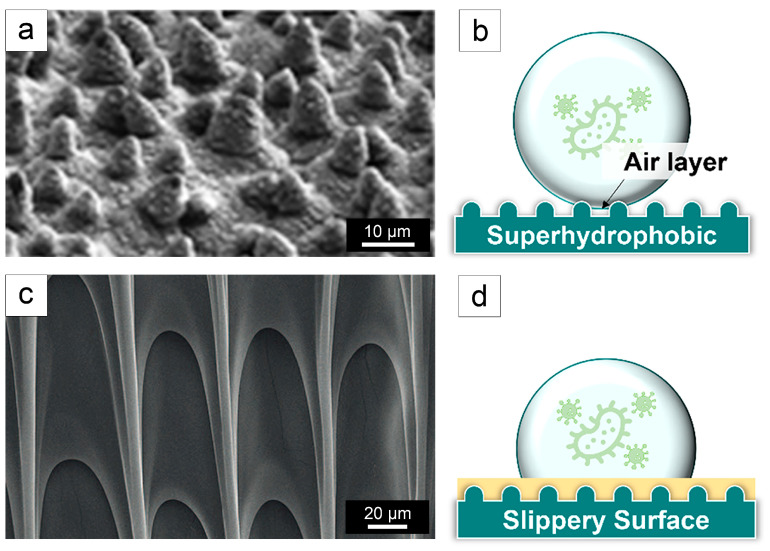
Natural antibacterial adhesion surfaces. (**a**) A scanning electron microscopy (SEM) image of a lotus leave [68]; (**b**) Schematic diagram of a super-hydrophobic surface [61]; (**c**) An SEM image of a *Nepenthes* [66]; and (**d**) Schematic diagram of a slippery surface [61]. Reproduced with permission from Refs. [61,66,68].

Natural surfaces primarily mitigate bacterial adhesion through their super-hydrophobic or super-smooth properties [61,64], as depicted in Figure 4. The lotus leaf surface exemplifies super-hydrophobic surfaces found in nature [56]. Its super-hydrophobicity results from a hierarchical micro/nano-scale structure, which also imparts self-cleaning properties [56], as shown in Figure 4a. Droplets on the lotus leaf are supported by micro/nano-structures, facilitating their movement and aiding in the removal of microorganisms [61]. This effect, known as the ‘lotus effect’, has been observed in various other plant surfaces. Typically, super-hydrophobic surfaces exhibit water contact angles exceeding 150°, combined with high surface roughness and low surface energy [64]. Moreover, an air layer between the suspended droplets and the super-hydrophobic surface reduces the contact area between bacteria and the surface, thereby decreasing bacterial adhesion and biofilm formation [61], as illustrated in Figure 4b. However, super-hydrophobic surfaces will lose their air layer when submerged in water for extended periods, leading to increased bacterial adhesion [65]. This phenomenon is undesirable for implants [65].

The concept of super-smooth surfaces resistant to bacterial contamination was inspired by the unique surface at the edge of the water jar of the *Nepenthes* plant. This surface achieves smoothness through a liquid layer on its micro-structure, which traps insects [66] (Figure 4c). Researchers have investigated this concept by examining the micro-structure of such surfaces. The surface is coated with a special liquid within its porous structure, forming a lubricious film that reduces microorganism adhesion [67] (Figure 4d). The liquid layer on super-smooth surfaces is analogous to the air layer on super-hydrophobic surfaces, effectively preventing bacterial adhesion and colonization [61].

Notably, neither super-hydrophobic surfaces nor super-smooth surfaces are bactericidal. Incorporating bactericidal properties into these surfaces represents a promising area for further exploration.

#### 2.1.3. Antibacterial Mechanisms of Natural Antibacterial Surfaces

The antibacterial mechanisms of natural surfaces are primarily influenced by the topographical parameters and surface wettability of micro/nano-structured surfaces [69]. Micro/nano-structures are crucial in determining both the antibacterial mode and the antibacterial ability [70]. Surfaces with micro/nano-structures, such as those found on cicada wings [33], dragonfly wings [36], and gecko skin [35], can directly kill bacteria. In contrast, surfaces like lotus leaves and *Nepenthes* achieve antibacterial effects by inhibiting bacterial colonization and biofilm formation [61,64].

The bactericidal effect of nano-pillar arrays with a high aspect ratio and high density, such as those on cicada and dragonfly wings, primarily relies on the penetration of the nano-pillar tips into the bacterial cell wall [33,37]. Upon interacting with bacteria, the nano-pillar arrays can rupture the bacterial cell wall [33,34].

In contrast, the bactericidal effect of high aspect ratio, low-density nano-spikes, such as those observed on gecko skin, particularly against smaller bacteria, may be attributed to a combination of forces [35]. These include surface adhesion between the bacteria and the sides of the spikes, as well as gravity acting on the bacteria [35]. These forces can cause stretching of the bacteria, leading to deformation or even rupture [38]. Such surfaces inhibit bacterial movement and biofilm formation [38]. Bacteria are primarily distributed on the micro/nano-structures rather than in the interstitial spaces [38].

Although antibacterial mechanisms may vary, bacteria generally adhere to micro/nano-structures, likely due to interactions between the micro/nano-structured surfaces and the bacteria [60,69]. The effectiveness of various biological micro/nano-structured surfaces against different bacteria can be attributed to differences in the thickness of bacterial cell walls [60,69]. Some bacteria may have cell walls that resist the stretching forces exerted by micro/nano-structures, thereby limiting the antibacterial effect [60,69].

In contrast, surfaces found on plants, such as lotus leaves, do not directly kill bacteria. Instead, their antibacterial effect primarily results from the super-hydrophobic nature of dense micro/nano-structures on their surfaces [71]. These micro/nano-structures suspend bacteria on the surface, creating an air layer between the bacteria and the surface [71]. This air layer hinders bacterial adhesion and colony formation [56].

Surface wettability primarily influences bacterial adhesion behavior. For example, *P. aeruginosa* tends to adhere to hydrophobic surfaces, while *S. aureus* adheres to hydrophilic surfaces [72]. This preference is determined by the surface energy of the bacteria [72]. Variations in micro/nano-structured surfaces result in differences in wettability [72]. However, there is no consensus on whether hydrophobic or hydrophilic surfaces are more resistant to bacterial adhesion.

The bactericidal efficacy of natural micro/nano-structured surfaces is effective only against specific bacteria. Antibacterial adhesion surfaces do not kill bacteria but rather hinder bacterial adhesion, which can make it more difficult for cells to adhere. Consequently, there is a need to further enhance the antibacterial capabilities of natural micro/nano-structured surfaces. Inspired by these natural micro/nano-structures, numerous artificial micro/nano-structured surfaces have been developed. By fine-tuning the topographical parameters of these surfaces, researchers have revealed their antibacterial mechanisms and improved their antibacterial efficacy.

### 2.2. Typical Artificial Antibacterial Surfaces

Naturally inspired antibacterial surfaces have provided innovative antibacterial options for orthopedic implants. Artificial surfaces exhibiting antibacterial effects similar to those of biological surfaces have been developed [35,37,57,73].

Wang et al. used molecular dynamics simulations to study the interaction between nano-structures and phospholipid bilayers, revealing that multiple nano-pillars produce a synergistic effect on antibacterial activity [74]. Their study demonstrated that nano-pillar arrays enhance the disruption of phospholipid bilayers on bacterial cell surfaces [74]. The deformation rate induced by nano-pillar on vesicles exceeded 100% [74]. Additionally, they found that nano-pillars with smaller diameters facilitate easier penetration of vesicles by the nano-pillar array [74]. Overall, nano-pillar arrays exhibit promising bactericidal and potentially virucidal effects [74].

Nano-pillar arrays with a high aspect ratio are a well-researched class of nano-structures. These arrays, derived from various materials using different biofabrication methods and featuring diverse parameters, have been extensively studied for their antibacterial properties.

#### 2.2.1. Factors Affecting the Antibacterial Effects of Nano-Pillar Arrays

Several factors can influence the antibacterial effects of nano-pillar arrays. Key contributing factors typically include height, radius, and density, which are often uniformly distributed across the arrays.

Ivanova’s group engineered nano-pillar array surfaces with uniform height, diameter, and regular alignment, which exhibit bactericidal properties against both Gram-positive and Gram-negative bacteria [75]. They have investigated the bactericidal effect of nano-pillar arrays with high aspect ratios by systematically varying the height of the nano-pillars [75]. During bacterial adhesion, bacteria induce deflection of the nano-pillars, which increases the stretching of the bacterial cell wall [75]. Within a certain range, the flexibility of the nano-pillars enhances the bactericidal effect [75]. However, when the aspect ratio is excessively high, the nano-pillars tend to cluster, leading to a reduction in bactericidal efficacy [75].

Waston et al. proposed a theoretical model based on surface energy to explain the interaction between bacteria and nano-pillar array surfaces [76]. They suggested that the surface tension resulting from the attraction between the nano-pillar array surface and the bacterial cell wall provides the energy needed to disrupt the bacteria [76]. By simplifying the bacterial cell wall to a thin plate and assuming that the bacterial cell radius is significantly larger than that of the nano-pillar, they disregarded the original curvature of the bacterial cell wall [76]. In scenarios where the bacterial wall thickness is infinitesimally thin, larger nano-pillar radii have been found to be more effective in killing bacteria with thicker or stiffer cell walls [76]. Notably, this study has revealed no strong correlation between bactericidal efficacy and nano-pillar spacing [76].

Tan et al. investigated the impact of nano-pillar array surface density on Gram-negative bacteria [77]. They found that when the spacing between nano-pillars is smaller than the size of bacterial cells, lower densities of nano-pillar arrays are more effective in reducing the initial bacterial adhesion within a certain range [77]. Changes in nano-pillar array density alters the interfacial energy between the nano-pillars and bacteria, thereby influencing the bactericidal effect of the surface [77]. Different interfacial energies create distinct driving forces for bacteria [77]. Specifically, at low nano-pillar array densities, the reduced contact area between bacteria and nano-pillars leads to increased tension on the bacterial membrane, resulting in a more pronounced bactericidal effect [77].

Xiao et al. demonstrated that the bactericidal effect of nano-pillar arrays correlates with both the density and radius of the nano-pillars [78]. They proposed that the equilibrium adhesion depth of bacteria along the nano-pillar, determined by minimizing the total free energy of the bacteria after adhesion, serves as a criterion for evaluating the bactericidal effect [78]. A greater adhesion depth increases the stretching of the bacterial membrane, thereby enhancing the bactericidal effect of the nano-pillar [78]. Theoretical analysis of total free energy suggested that the nano-pillar must meet certain radius requirements to ensure effective bacterial adhesion [78]. Furthermore, an increase in the density of cylindrical nano-pillar arrays leads to a greater bacterial adhesion depth [78]. Within a certain range, a cylindrical nano-pillar array characterized by high density and small radius exhibits superior bactericidal efficacy [78]. These factors collectively determine the bactericidal effect [78].

Ishak et al. fabricated nano-pillar arrays with varying aspect ratios and densities and investigated their impact on bacterial metabolic activity and bacterial membranes [79]. They found that nano-pillar array density has a more pronounced effect on bacterial activity, with lower activity observed on surfaces with higher nano-pillar densities [79]. The impact of nano-pillar arrays on bacterial membranes primarily manifests as membrane damage and deformation [79]. Notably, nano-pillar arrays with high aspect ratios and densities cause greater membrane damage, especially to Gram-negative bacteria, with Gram-positive bacteria showing less than 10% damage [79]. Bacterial membrane deformation between nano-pillars occurs in three main forms: flat, inward, and outward deformation [79]. This deformation induces stress, thereby reducing bacterial activity and enhancing the bactericidal properties of the nano-pillar arrays [79].

In summary, mechanical forces are crucial to the bactericidal action of nano-pillar arrays against bacteria. This mechanism involves various factors, including the deformation force during bacterial cell adhesion, the shear force exerted by bacterial movement, and the wettability of the nano-pillar arrays. These elements synergistically contribute to the specific bactericidal effects of nano-pillar arrays.

#### 2.2.2. Antibacterial Mechanism of Nano-Pillar Arrays

**Figure 5:**
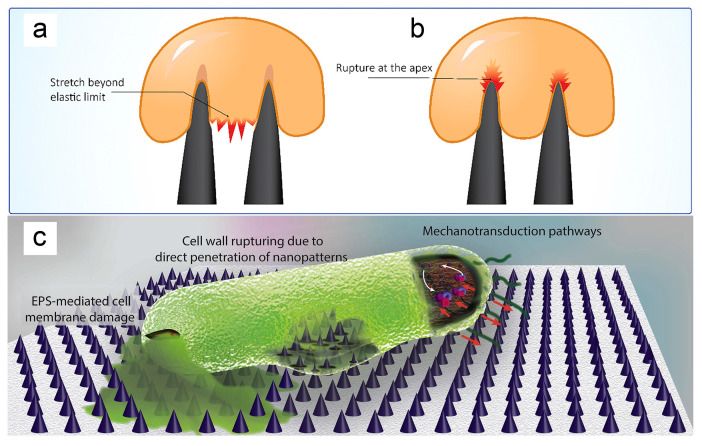
Bactericidal mechanisms of micro/nano-structures. (**a**) The bacterial membrane suspended between two nano-pillars is stretched beyond its elastic limit [60]; (**b**) Bacterial membranes at the tips of nano-pillars are prone to be ruptured [60]; and (**c**) Movement of bacteria on the micro/nano-structured surface leads to bacterial rupture which is lethal to bacteria [69]. EPS: Extracellular polymeric substances. Reproduced with permission from Refs. [60,69].

Previous studies have shown that the bactericidal mechanism of nano-pillar arrays is multi-faceted, with varying perspectives on their mechanical effects. Fundamentally, these effects arise from interactions between micro/nano-structures and bacterial membranes. The prevailing view is that mechanical bactericidal effects occur when the bacterial membrane, adhering to the micro/nano-structured surface, is stretched beyond its elastic limit, leading to rupture and bacterial death [60], as shown in Figure 5. However, there is debate over the precise location of membrane deformation. Pogodin et al. suggested that lethal stretching occurs in the region suspended between the protrusions [59,60], as depicted in Figure 5a. Conversely, Velic et al. proposed that maximum deformation happens where the bacterial membrane contacts the tips of the nano-protrusions [60,80], as illustrated in Figure 5b.

Salatto et al. fabricated a nano-pillar array surface capable of both killing bacteria and releasing dead bacteria [81]. They conducted molecular dynamics simulations to investigate the interaction between the phospholipid bilayer of *E. coli* and cylindrical hydrophilic nano-pillars [81]. By modulating the interaction strength, they demonstrated that the bactericidal effect of the nano-pillar arrays primarily results from the attraction of the nano-pillars to the bacterial membrane [81]. Localized high tension in the high-curvature region at the edge of the nano-pillars leads to rupture of the bacterial membrane [81]. Additionally, adsorption of the nano-pillars to the hydrophilic head of the phospholipid bilayer induces re-arrangement of the phospholipid bilayer and deformation of the bacteria [81]. Once bacterial deformation exceeds a certain limit, membrane rupture occurs, resulting in bacterial death [81]. Moreover, if the attraction force is sufficiently high, even shorter nano-pillars can exhibit bactericidal effects [81].

Alternative perspectives suggested that the shear force generated by bacterial movement after adhesion to nano-pillar arrays is the primary cause of bacterial death [69], as illustrated in Figure 5c. Strong adhesion of extracellular polymeric substances (EPS) to nano-patterns and subsequent attempts by bacteria to move away from these surfaces can result in bacterial membrane damage [69].

Bandara et al. [36] and Jindai et al. [82] proposed that the bactericidal effect of nano-pillar arrays results from the shear force generated by the movement of bacteria adhered to the nano-pillar surface. The motility of bacteria is crucial to the bactericidal effects of these arrays [36,82]. Bandara et al. demonstrated that during the initial stages of bacterial adhesion to high nano-pillar arrays, the tall nano-pillars bend, leaving the bacterial membrane intact [36]. However, as bacteria move across the surface, their membranes stretch and the shear force exerted by the nano-pillars causes membrane rupture [36]. Similarly, Jindai et al. found that membrane damage is more pronounced in motile bacteria compared to non-motile bacteria [82]. As a result, nano-pillars are less effective at killing non-motile bacteria [82].

Researchers have designed and fabricated nano-pillar arrays with varying topographical parameters across different materials, exploring how these factors influence the antibacterial effects. However, the bactericidal efficacy of biomimetic micro/nano-structured surfaces still falls short of achieving both high efficiency and broad applicability. Relying solely on the mechanical bactericidal properties of micro/nano-structured surfaces appears insufficient for Ti implant surfaces.

## 3. Photocatalytic Antibacterial Ability of TiO_2_

The natural oxide layer on Ti surfaces, TiO_2_, offers several advantages, including low cost, excellent biocompatibility, and low toxicity [83]. TiO_2_ is widely used as a photocatalyst [84]. Recently, the photocatalytic bactericidal potential of nano-TiO_2_ has been extensively investigated across various fields. Unlike chemical-based methods, the photocatalytic action of TiO_2_ involves active stimulation, making it a promising option for Ti implants [84]. This approach avoids reliance on chemicals, contributing to the growing interest in TiO_2_ for implant applications [84]. TiO_2_ has been shown to enhance the biological inertness and cytocompatibility of Ti implants [45]. Additionally, various TiO_2_ micro/nano-structured surfaces possess unique properties, such as facilitating bone regeneration and repair [54], influencing the migration, extension, proliferation, and differentiation of stem cells [84], and exhibiting significant antibacterial activity [85].

**Figure 6:**
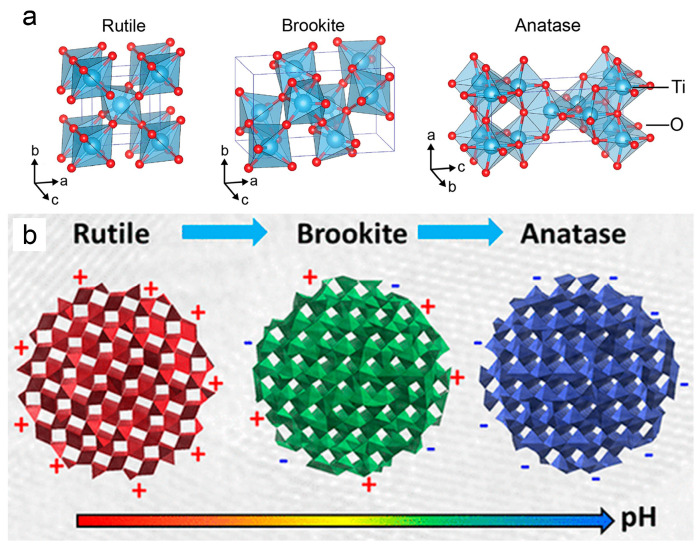
(**a**) Crystal structures of TiO_2_: rutile, anatase, and brookite [86]; and (**b**) The influence of pH on TiO_2_ crystal structure [89]. Reproduced with permission from Refs. [86,89].

TiO_2_ exhibits three primary crystal structures: rutile, anatase, and brookite [86]. These structures typically consist of six oxygen atoms surrounding a Ti atom, forming an octahedron [86]. Rutile and anatase have tetragonal structures and are stable, making them common in TiO_2_-related applications [86], as shown in Figure 6a. In contrast, brookite has an orthorhombic structure, which is less stable and often a by-product of preparing other crystalline phases [87]. Brookite’s instability, especially at higher temperatures, limits its practical use [88]. Among the three structures, anatase and brookite can transform into rutile through high-temperature calcination processes [54]. Conversely, rutile and brookite can transform into anatase under high pH conditions [89], as illustrated in Figure 6b. The stability of TiO_2_ crystal structure under various environmental conditions is crucial for research [89].

**Figure 7:**
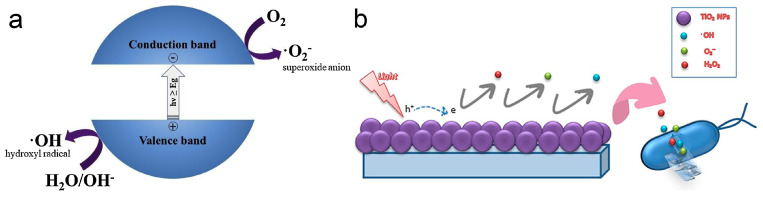
(**a**) Photocatalytic performance of TiO_2_ [44]; and (**b**) Photocatalytic antibacterial mechanism of TiO_2_ [45]. NPs: Nanoparticles. Reproduced with permission from Refs. [44,45].

TiO_2_ is a semiconductor material with a band gap ranging from 3.0 to 3.2 eV, providing specific electronic and photocatalytic properties [84,90]. Its photocatalytic behavior relies on electron migration [44]. When exposed to light, electrons in the valence band of TiO_2_ are excited to higher energy levels in the conduction band [44]. This excitation generates electron–hole pairs, leading to the production of two types of ROS—hydroxyl radicals and superoxide ions—on the material’s surface [44], as illustrated in Figure 7a.

The photocatalytic properties of TiO_2_ are widely applied in sterilization and disinfection processes. Hydroxyl radicals produced through photocatalysis are highly effective at deactivating Gram-negative bacteria, whereas superoxide ions demonstrate strong bactericidal activity against Gram-positive bacteria [44]. This effectiveness is due to the capability of photocatalytically generated ROS to damage bacterial membranes [44,45]. The antibacterial mechanism of TiO_2_ is illustrated in Figure 7b [45].

The photocatalytic behavior of TiO_2_ is significantly influenced by its crystal structure and form [54]. Rutile, while exhibiting faster recombination of electron-hole pairs, shows photocatalytic properties even under partial visible light [86]. In contrast, anatase demonstrates superior photocatalytic activity; however, its band gap energy of 3.2 eV and absorption edge at 385 nm restrict its photocatalytic performance to the ultraviolet (UV) light region [44,83]. Studies suggested that anatase tends to be more stable and dominant among nanomaterials [91]. Some investigations also indicated that brookite may exhibit higher photocatalytic activity compared to anatase and rutile phases [86,87]. Nevertheless, its complex preparation and purification processes limit its practical application [86,87].

The use of nano-TiO_2_ in the biomedical field has attracted considerable attention. Unlike most metal NPs, which exhibit antibacterial activity through the release of metal ions and may cause moderate cytotoxicity [92], nano-TiO_2_ does not release Ti^4^⁺ ions [93]. Instead, its antibacterial activity primarily stems from its impact on bacterial cell membranes and the generation of ROS [92,93]. Research indicated that nano-TiO_2_ is effective against a variety of bacteria, including *E. coli*, *S. aureus*, and MRSA [94,95]. Furthermore, nano-TiO_2_ has shown efficacy against biofilms formed by certain bacteria [96].

Besinis et al. demonstrated that nano-TiO_2_ can partially inhibit the growth of *Streptococcus pyogenes* (*S. pyogenes*) [97]. Sohm et al. explored the bactericidal mechanism of nano-TiO_2_ against *E. coli* under dark conditions and proposed that nano-TiO_2_ induces depolarization of bacterial cell membranes and loss of membrane integrity [98]. Jesline et al. found that nano-TiO_2_ exhibits antibacterial activity against MRSA, and this activity operates independently of any antibiotics [99].

**Figure 8:**
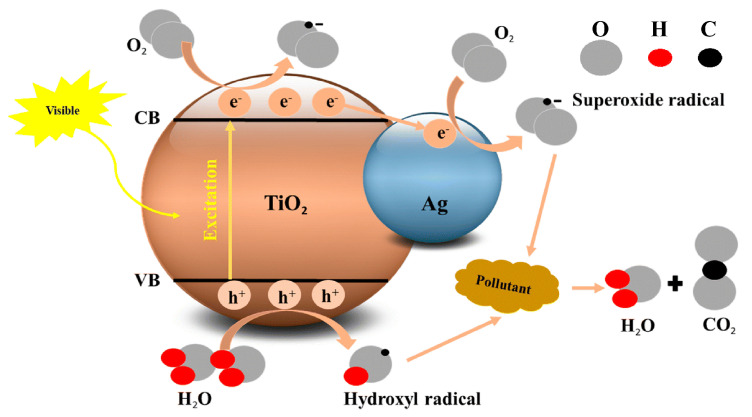
Schematic of photocatalytic activity of Ag NPs-doped TiO_2_ [101]. CB: Conduction Band. VB: Valence Band. Reproduced with permission from Ref. [101].

While the photocatalytic properties of nano-TiO_2_ enhance bactericidal activity, TiO_2_ primarily produces ROS under UV excitation, which limits its use in biomedicine [100,101]. Methods such as metal ion or compound doping can shift TiO_2_’s light absorption range toward visible light, expanding its applicability [100,101]. This doping generally involves metal ions replacing Ti^4+^ ions in TiO_2_ or forming heterostructures with TiO_2_ [101,102,103]. For example, Ag NPs doped into TiO_2_ not only reduce the recombination of electron–hole pairs but also shift the light absorption range toward the visible spectrum [101], as shown in Figure 8. Ag acts as an electron trap, capturing electrons from the conduction band of TiO_2_ and thereby improving photocatalytic performance [101]. Jiang et al. developed self-doped blue TiO_2_ containing Ti^3+^ ions and oxygen vacancies, which exhibit excellent photocatalytic performance under visible light [104]. Liang et al. prepared Bi_2_S_3_-TiO_2_ composites with various topographies, finding that Bi_2_S_3_ modification improves the light absorption of TiO_2_ [105]. Other dopants such as Bi_4_Ti_3_O_12_, SrTiO_3_, and Selenium (Se) have been used to enhance the photocatalytic and antibacterial properties of different TiO_2_ forms [102,103,106,107,108]. Additionally, colored TiO_2_ materials exhibit better light absorption, which extends the photocatalytic active range of TiO_2_ [104,109]. This approach represents a promising research direction for improving photocatalytic antibacterial properties.

Additionally, the photocatalytic properties of TiO_2_ are highly dependent on its size and morphology [44]. Multiscale structures contribute to the enhancement of TiO_2_’s photocatalytic activity [48], and one-dimensional sharp TiO_2_ nanorods exhibit superior antibacterial capacity [48,110]. Two-dimensional TiO_2_ micro/nano-structures increase the effective surface irradiation area [111]. Both TiO_2_ nano-pillar and nanotube arrays enhance electron transfer and light scattering, thus improving light-harvesting efficiency [112]. Furthermore, the internal light scattering within TiO_2_ nanotube arrays further enhances their light-harvesting efficiency [112].

Consequently, integrating the photocatalytic antibacterial properties of TiO_2_ with the mechanical bactericidal effects of biomimetic micro/nano-structures has become a focal point of research. The widespread development of TiO_2_ micro/nano-structured surfaces emerges as a promising strategy to enhance the antibacterial efficacy of Ti implants.

## 4. Biofabrication Methods: Integrating TiO_2_ with Micro/Nano-Structured Surfaces

Researchers have employed various biofabrication methods to prepare TiO_2_ micro/nano-structures on Ti surfaces, including electrochemical anodic oxidation (EAO), chemical vapor deposition (CVD), template method, sol-gel method, laser ablation, electrostatic spinning, and hydrothermal method [88,113,114,115,116,117,118,119,120,121,122,123,124,125,126]. The advantages and drawbacks of different biofabrication methods are shown in Table 1.

Hydrothermal method involves the crystallization of substances in an aqueous solution under high-temperature and -pressure conditions [112]. Typically conducted in a vessel with controlled temperature and pressure, the process operates at temperatures exceeding 100 °C, causing the solvent to vaporize, while the pressure inside the vessel surpasses atmospheric pressure [50]. Teflon-lined autoclaves and corresponding stainless-steel shells are commonly used for this synthesis. Initially employed for small particle synthesis in the ceramics industry, hydrothermal methods later gained popularity for nano-material synthesis. Depending on the composition of the reaction solution, hydrothermal method can be classified into alkaline and acidic methods. The selection of the Ti source is critical and generally involves Ti alcohol salts [127], TiO_2_ powder [128], or Ti foil [129]. Below, we outline the preparation of TiO_2_ micro/nano-structured surfaces using various hydrothermal methods, highlighting TiO_2_ topography and crystal structure, as summarized in Table 2.

### 4.1. Alkaline Hydrothermal Methods

Alkaline hydrothermal method involves the use of sodium hydroxide (NaOH) solution to establish an alkaline environment. The process typically begins by reacting a Ti source with a NaOH solution of specific concentration under high-temperature and -pressure conditions [143]. The resultant product is then subjected to a pickling process, followed by annealing and additional treatments to obtain the final sample [143]. The fundamental principles of the alkaline hydrothermal method reaction are as follows [143]:

3TiO_2_ + 2NaOH → Na_2_Ti_3_O_7_ + H_2_O (1)

Na_2_Ti_3_O_7_ + 2HCl → H_2_Ti_3_O_7_ + 2NaCl (2)

H_2_Ti_3_O_7_ → 3TiO_2_ + H_2_O (3)

This method involves: (1) the hydrothermal generation of titanate from NaOH and a Ti source in an alkaline environment; (2) ion exchange between the titanate and an acid solution during pickling to produce titanic acid; and (3) the annealing of titanic acid to form TiO_2_.

The alkaline hydrothermal method is widely used to fabricate various TiO_2_ nano-structures, including nano-tube films [130,131], nano-wire arrays [128,133], nano-pillar arrays [129,136,137], etc.

Luo et al. [130] and Sun et al. [131] employed NaOH as the hydrothermal solvent to prepare one-dimensional TiO_2_ nano-tube films on Ti sheets and foils. Luo et al. utilized plasma electrolytic oxidation to create a seed layer on Ti plates, followed by the synthesis of single-crystal anatase TiO_2_ nano-tube films on this seed layer [130]. In contrast, Sun et al. produced rutile TiO_2_ nano-tube films via hydrothermal method on oxidized Ti foil [131]. The one-dimensional nano-tube films demonstrates enhanced surface photocatalytic activity [131]. Luo’s nano-tube films were formed by the bending of nanosheets [130], whereas Sun et al. observed that NPs dissolved into continuous ridge structures, eventually forming one-dimensional nano-tubes by passing through the nano-sheets [131].

Zhang et al. fabricated TiO_2_ nano-wire arrays in situ on the TiO_2_ NP compacts [128]. The resulting arrays exhibited a combination of randomly distributed outer layers and vertically arranged inner layer nano-wires, with the nano-wires being highly wound [128]. These arrays can support cell attachment and proliferation and exhibit good biocompatibility [128]. Jaggessar et al. synthesized nano-wire arrays with a random structural layout on Ti sheets, adjusting various parameters to achieve a nano-wire network with excellent mechanical properties [133]. This network structure is effective against *S. aureus* [133].

Zhang et al. produced nano-pillar arrays with a length of approximately 1 μm and a diameter of 40–50 nm [129]. Later, under varying conditions, Zhang et al. synthesized nano-pillar arrays with a length of 1.35 μm and a diameter of 50 nm [136]. Similarly, He et al. optimized the reaction conditions to create nano-pillar arrays with a diameter of approximately 45 nm [137]. Notably, in He et al.’s study, the orientation of the nano-pillar arrays was not perfectly vertical [137].

Gao et al. prepared TiO_2_ nano-spikes on Ti sheets, featuring needle-like nano-fibers with a diameter of approximately 47 nm and a height of about 4 μm [51]. This nano-spike surface significantly deforms bacteria [51]. TiO_2_ nano-spikes prepared by Huo et al. on Ti plates also exhibit notable bactericidal ability [52].

Additionally, Li et al. developed TiO_2_ nano-structured arrays, including nano-sheets, nano-belts, and nano-wire films, on Ti plates [53]. During the hydrothermal reaction, sodium titanate crystal nuclei initially formed on the Ti plates [53]. Subsequently, Ti atoms dissolved and recrystallized on the surface of these sodium titanate nuclei, resulting in anatase TiO_2_ [53]. Variations in NaOH concentration, hydrothermal temperature, and reaction duration influence the nucleation, dissolution, and recrystallization processes, leading to distinct surface topographies [53].

Moreover, Zhang et al. used Ti foil as the Ti source and reacted it with NaOH and mannitol to produce sodium titanate [132]. This was followed by heat treatment to prepare anatase CMTFs [132]. The inclusion of mannitol led to the formation of chiral twisted NPs, resulting in mesoporous TiO_2_ films with multiple chiral features [132].

Furthermore, the alkaline hydrothermal method using tetramethylammonium hydroxide (TMAOH) as the reaction solution has garnered attention. This method eliminates the need for subsequent annealing, allowing for the direct synthesis of single-crystal TiO_2_ nano-shovel arrays [134,135].

### 4.2. Acidic Hydrothermal Methods

In acidic hydrothermal method, hydrochloric acid (HCl) is commonly used as the reaction medium. This method is more complex compared to alkaline hydrothermal method due to the intricate reaction mechanisms involved. The specific reaction processes can vary depending on the Ti sources employed. Acidic hydrothermal method predominantly yields TiO_2_ nano-pillar arrays. The principle behind the preparation of TiO_2_ nano-pillar arrays via acidic hydrothermal method is illustrated by the following equations [144,145,146]:

2Ti + 6HCl → 2TiCl_3_ + 3H_2_ (4)

Ti^3+^ + H_2_O → TiOH^2+^ + H^+^ (5)

TiOH^2+^ → O_2_^−^ + Ti(IV) oxo species → TiO_2_ (6)

The acidic hydrothermal method involves two primary processes: dissolution and growth. Initially, Ti dissolves upon reaction with an acidic solution under high-temperature and -pressure. Subsequently, Ti ions undergo a complex series of reactions in water, forming complexes that eventually grow into TiO_2_ nano-pillars [144,145,146]. Ti(IV) oxo species are recognized as intermediate growth units in the formation of TiO_2_ [144].

The acidic hydrothermal method is commonly utilized to fabricate TiO_2_ nano-pillar arrays on fluorine-doped tin oxide (FTO) conductive glass. This method primarily aims to enhance the photochemical properties of photocatalytic materials for applications such as solar cells [127,139]. Researchers have employed various precursor solutions and investigated hydrothermal reaction conditions to optimize the growth of TiO_2_ nano-pillar arrays.

For instance, Khizir et al. prepared rutile TiO_2_ nano-pillars on FTO using TiCl_4_ and HCl as the reaction solution [138]. Prathan et al. employed a mixture of Ti butoxide and HCl to prepare TiO_2_ nano-pillar arrays on FTO, incorporating TiO_2_ seed layers [127]. Ben Naceur et al. used HCl and Ti isopropoxide to produce rutile TiO_2_ nano-pillar arrays on FTO and investigated the effect of hydrothermal reaction duration on the properties of these arrays [139]. Meshesha et al. prepared rutile TiO_2_ nano-pillar and nano-wire arrays on FTO using HCl and Ti butoxide, determining the optimal reaction temperature for the synthesis of TiO_2_ nano-pillars and nano-wires [140].

Moreover, TiO_2_ nano-pillar arrays have also been synthesized via the acidic hydrothermal method on various substrates. Wang et al. developed controllably patterned TiO_2_ nano-pillar arrays on Si substrates using TiO_2_ seed layers and a precursor solution composed of HCl, isopropanol, tetrabutyl titanate, and ethyl acetate [141]. TiO_2_ nano-pillar arrays have also been prepared on HCl-soaked Ti foils with a hydrothermal reaction solution comprising KCl and HCl [142]. Additionally, TiO_2_ nano-tube arrays have been fabricated on untreated Ti foils using a mixture of KCl and NaOH, which demonstrates the promoting effect of Cl⁻ ions on TiO_2_ nano-tube growth [142].

### 4.3. Comparison of Alkaline and Acidic Hydrothermal Methods

**Figure 9:**
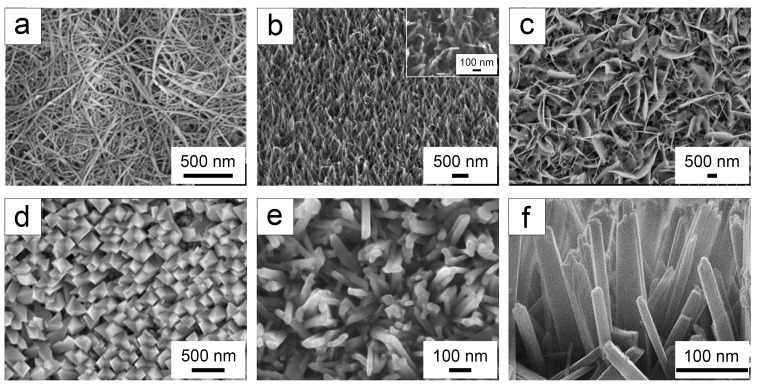
Micro/nano-structured surfaces prepared by alkaline (**a**–**e**) and acidic (**f**) hydrothermal methods. (**a**) Nano-tube films [130]; (**b**) Nano-wire arrays [133]; (**c**) Nano-sheet arrays [53]; (**d**) Nano-shovel arrays [134]; (**e**) Nano-pillar arrays [137]; and (**f**) Nano-pillar arrays [140]. Reproduced with permission from Refs. [53,130,133,134,137,140].

From Table 2, it is evident that hydrothermal methods enable the fabrication of TiO_2_ nano-structured surfaces with various crystal structures. Specifically, the alkaline hydrothermal method produces TiO_2_ nano-tube films, TiO_2_ nano-wire arrays, and TiO_2_ nano-pillar arrays, which differ primarily in topography, as illustrated in Figure 9a–e. In contrast, the acidic hydrothermal method is predominantly used to generate TiO_2_ nano-pillar arrays, as shown in Figure 9f. Notably, TiO_2_ nano-pillar arrays produced via acidic hydrothermal methods exhibit a vertically aligned orientation, while those created using alkaline hydrothermal methods display a more random arrangement. Regarding substrates, the alkaline hydrothermal method typically uses Ti sheets or Ti foils, which also serve as the Ti source for the reaction. Conversely, the acidic hydrothermal method accommodates a wider range of substrates, including FTO, Ti sheets, and Si substrates. Additionally, acidic hydrothermal methods generally require extra steps to prepare a TiO_2_ seed layer on the substrate. 

The crystal structure and topography of TiO_2_ are significantly influenced by various factors, including the Ti source, acid or alkali concentration, hydrothermal duration, hydrothermal temperature, and annealing temperature. Despite extensive research, a universal explanation for these phenomena remains elusive. Understanding these factors is crucial for developing TiO_2_ micro/nano-structured surfaces suitable for various applications. In implant-related fields, alkaline hydrothermal methods are particularly advantageous due to their ability to use Ti as the substrate, which allows for a wide range of topographical options. Numerous TiO_2_ micro/nano-structured surfaces have been successfully fabricated on Ti substrates using alkaline hydrothermal methods, and their antibacterial properties and application potential have been thoroughly evaluated.

## 5. Antibacterial Ability of TiO_2_ Biomimetic Micro/Nano-Structured Surfaces

Naturally inspired micro/nano-structured surfaces demonstrate promising bactericidal and antibacterial adhesion properties. Concurrently, extensive research has explored the cytocompatibility of TiO_2_ and its photocatalytic antibacterial properties. The development of biomimetic micro/nano-structures on implant surfaces using hydrothermal methods represents a crucial advancement in implant surface modification. TiO_2_ nano-pillar arrays, a prominent biomimetic micro/nano-structured surface, are particularly noted for their effective physical and mechanical sterilization properties, making them a popular choice among researchers. Consequently, research into TiO_2_ nano-pillar arrays for antibacterial applications is rapidly expanding.

### 5.1. Mechanical Antibacterial Ability

**Figure 10:**
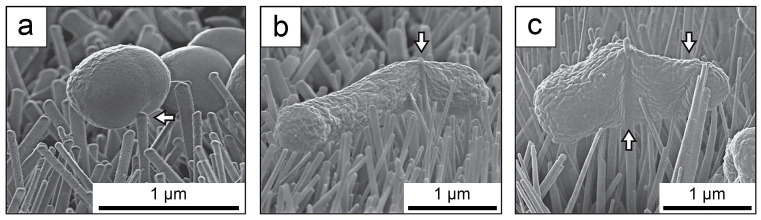
Bacterial morphologies on TiO_2_ nano-pillar arrays after 3 h of static incubation. SEM images of (**a**) *S. aureus* [147]; (**b**) *E. coli* [147]; and (**c**) *K. pneumoniae* [147]. The white arrows in (**a**–**c**) indicate the areas of bacterial cell wall deformation caused by nano-pillars. Reproduced with permission from Ref. [147].

The mechanical antibacterial effect of TiO_2_ nano-pillar arrays is primarily attributed to the stretching and tearing of bacterial membranes, which leads to the rupture of bacterial cells [38,147], as illustrated in Figure 10. TiO_2_ nano-pillar arrays demonstrate substantial mechanical bactericidal effects against *S. aureus*, *E. coli*, and *Klebsiella pneumoniae* (*K. pneumoniae*) [147]. However, research on the mechanical antibacterial effects of TiO_2_ nano-pillar arrays is still limited, and the antibacterial efficacy attributed solely to their topography is not well established. Systematic investigations into how factors such as height, diameter, and gap distance influence antibacterial performance are lacking.

For instance, Cao et al. prepared a TiO_2_ nano-pillar array with a diameter of approximately 50–70 nm, which exhibited a bactericidal efficiency of 37% against *Staphylococcus epidermidis* (*S. epidermidis*) over a short period [148]. However, this array lacks long-term antibacterial effectiveness, as biofilm formation has been observed on the surface after 6 days of incubation [148]. Rao et al. fabricated TiO_2_ nano-pillar arrays with a diameter of about 100 nm on Ti alloy, noting obvious bacterial adhesion under dark conditions during both short and long-term incubation [149]. Pang et al. synthesized prismatic TiO_2_ nano-pillar arrays with an average diameter of approximately 200 nm on Ti plates [150]. After 24 h of incubation, these surfaces demonstrated 50–60% inhibition of *E. coli* and *S. aureus* [150]. Cheng et al. prepared uniformly distributed TiO_2_ nano-pillar arrays on Ti plates with heights ranging from 80 to 180 nm [151]. These arrays induce irregular changes in bacterial morphology but demonstrate a limited long-term antibacterial effect [151]. Jaggessar et al. produced TiO_2_ nano-pillar arrays with an average height of approximately 307 nm on a Ti alloy surface, observing a 46% reduction in bacterial viability after 18 h of incubation with *S. aureus*, compared to bare Ti surfaces [152]. Guan et al. synthesized TiO_2_ nano-pillar arrays with diameters ranging from 50 to 100 nm and heights of 1 to 2 μm on Ti plates [153]. They observed alterations in bacterial morphology after 24 h of incubation and a significant reduction in bacterial presence compared to bare Ti surfaces after 48 h [153]. Maher et al. fabricated TiO_2_ nano-pillar arrays with an average height of approximately 550 nm and a gap distance of about 130 nm on Ti orthopedic implants [154]. This configuration resulted in only a slight reduction in bacterial activity after 5 h of incubation [154].

Overall, most studies suggest that the mechanical antibacterial effect of hydrothermally prepared TiO_2_ nano-pillar arrays on medical Ti plates or Ti alloys is limited [106]. The mechanical bactericidal effect alone is insufficient for providing either short-term or long-term antibacterial properties. The impact of various topographical parameters on antibacterial efficacy remains underexplored, making it challenging to isolate the effect of a single parameter. The mechanical bactericidal effect of these micro/nano-structured surfaces results from the combined influence of multiple topographical factors. It is important to note that the mechanical antibacterial effect of TiO_2_ nano-pillar arrays is persistently passive and does not contribute to the development of bacterial resistance. Consequently, researchers have investigated additional antibacterial strategies, such as the photocatalytic bactericidal effect of TiO_2_, to enhance the antibacterial efficacy of TiO_2_ nano-pillar arrays.

### 5.2. Photocatalytic Antibacterial Ability

TiO_2_ functions as a stable semiconductor photocatalyst, primarily activated under UV light, which generates ROS upon irradiation [45]. However, UV light exposure can also harm cells, limiting the direct application of TiO_2_ for antibacterial photocatalysis. Additionally, the rapid recombination of charge carriers in TiO_2_ reduces its photocatalytic efficiency [84]. To address these issues, researchers have focused on enhancing TiO_2_ ability to absorb visible or near-infrared (NIR) light. This enhancement is achieved through topographical and structural modifications [155,156,157] or doping with metal ions [101,104] or compounds [105]. TiO_2_ biomimetic micro/nano-structured surfaces combine the mechanical bactericidal properties of biomimetic structures with the photocatalytic antibacterial capabilities of TiO_2_, offering significant advantages.

**Figure 11:**
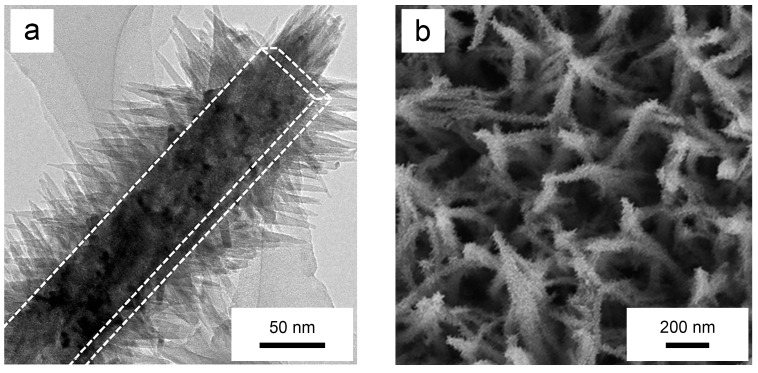
(**a**) A transmission electron microscopy (TEM) image of a single branched TiO_2_ nano-pillar [156]; and (**b**) A field emission SEM (FESEM) image of branched TiO_2_ nano-pillars [157]. Reproduced with permission from Refs. [156,157].

Yang et al. fabricated regularly arranged TiO_2_ nano-pillar arrays on Ti alloy surfaces using a bi-directional hydrothermal approach involving both alkaline and acidic conditions [155]. These TiO_2_ nano-pillars have exhibited varying valence states of Ti along their height, demonstrating antibacterial properties against both *E. coli* and *S. aureus* under standard conditions [155]. The nano-structure has enhanced the NIR absorption of the surfaces, significantly improving antibacterial efficacy [155]. Evaluation of the methylene blue degradation rate under NIR light (808 nm) exposure has revealed notable photocatalytic activity in the NIR region [155]. The surface has shown substantial antibacterial efficacy against both bacterial strains, with in vitro antibacterial rates exceeding 95%, surpassing those of Ti plates [155]. Analysis of intracellular ROS content has indicated that the photocatalytic effect predominantly drives the antibacterial performance [155]. Similarly, single-crystal branched TiO_2_ nano-pillar arrays prepared by Cho et al. [156] and Wu et al. [157], as shown in Figure 11, have exhibited enhanced photocatalytic properties.

Zhang et al. synthesized TiO_2_ nano-pillar arrays on Ti plates, with an average diameter of 35 nm and an average height of 1.35 μm [158]. Initially, the bactericidal efficacy of these nano-pillars was marginal, with inhibition rates against *S. aureus* and *E. coli* of 4.8% and 10.8%, respectively [158]. However, after 10 min of NIR light irradiation at 808 nm with a power density of 0.6 W/cm^2^, the inhibition rates increased to 19.2% and 24.3% [158]. Another study demonstrated that TiO_2_ nano-pillar arrays on Ti foil could effectively eradicate bacterial biofilms when irradiated with 808 nm NIR light at 0.8 W/cm^2^ for just 15 min [129]. Zhang suggested that the antibacterial effect is primarily due to ROS generated by NIR light irradiation, which alters bacterial membrane permeability [120]. The antibacterial efficiency further improves with increased power and longer irradiation duration [129].

## 6. Application Prospects and Future Directions of TiO_2_ Biomimetic Micro/Nano-Structured Surfaces

Orthopedic and dental implants are crucial in modern medicine. Implants made from Ti and its alloys, particularly those featuring TiO_2_ nano-pillar arrays, represent a promising direction for future development [79,147,159]. However, they face ongoing challenges, including bacterial infections and incomplete osseointegration [160,161]. To overcome these challenges, strategies such as doping or modifying TiO_2_ nano-pillar array surfaces have been explored. As previously discussed, altering micro/nano-structure topography parameters has minimal impact on improving antibacterial performance [106]. Moreover, doping elements or compounds can enhance the photocatalytic properties of TiO_2_ nano-pillar arrays, enabling effective antibacterial activity under NIR or visible light [101,102,103].

Although this discussion primarily has focused on the antibacterial strategy of TiO_2_ nano-pillar arrays, other topographies, such as nano-sheets and nano-tubes, also warrant further exploration. These structures possess unique properties and performance characteristics that could provide valuable insights for enhancing antibacterial ability of implants [115,162,163].

**Figure 12:**
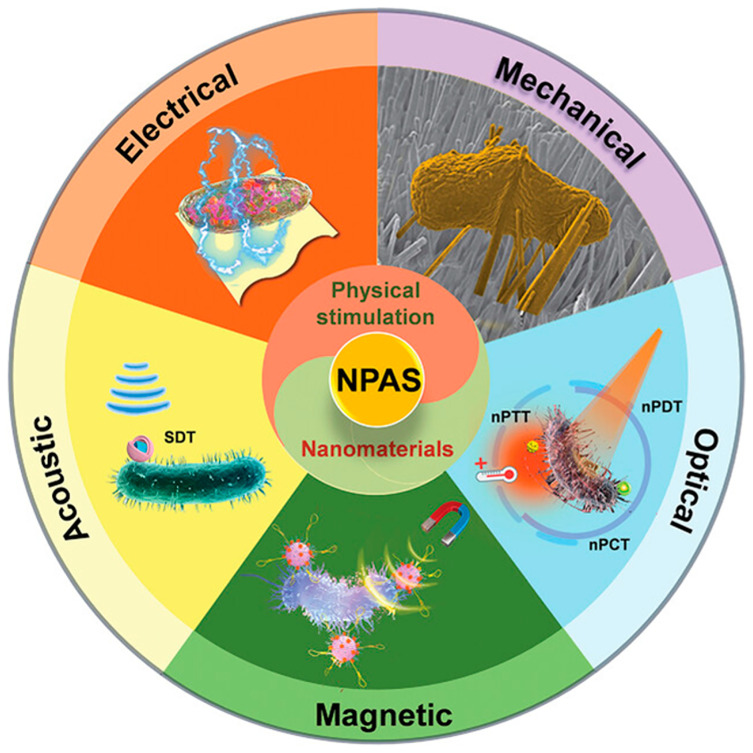
Nano-physical antibacterial strategies (NPAS) categorized by their mode of physical stimulation, including mechanical, optical, magnetic, acoustic, and electrical signals [165]. Mechanical antibacterial strategy: To highlight the morphology change of the bacterium, the false color has been used in this SEM image. SDT: Sonodynamic Therapy. nPTT: Nano-photothermal Therapy. nPDT: Nano-photodynamic Therapy. nPCT: Nano-photocatalytic Therapy. Reproduced with permission from Ref. [165].

Combined antibacterial strategies offer enhanced efficacy, comprehensive performance, and increased safety for improving implants [21,106,162]. The integration of micro/nano-structure based physical/mechanical/chemical bactericidal effects has demonstrated promising results both in vitro and in short-term in vivo studies [164]. Additionally, the combination of nanomaterials with physical stimulation modalities—including mechanical, optical, magnetic, and acoustic signals—represents a promising therapeutic approach for addressing bacterial antibiotic resistance [165], as illustrated in Figure 12.

TiO_2_ micro/nano-structured surfaces with mechanical, chemical, and photocatalytic antibacterial effects represent a dominant focus of current research [166]. Emerging treatments, such as electrical stimulation sterilization, magnetic sterilization, and sonodynamic therapy, also warrant further investigation. Electrical stimulation has been employed to alter cell morphology and enhance immune cytokine secretion [167,168]. Bioelectrical methods have potential for bacterial eradication [168]. Magnetic sterilization functions by denaturing bacterial proteins and inducing structural damage through generated electrical currents [168]. Acoustic sterilization is primarily achieved via sonodynamic therapy, where ultrasound excites an acoustic sensitizer to produce electron transfer, which subsequently reacts with water and oxygen to generate ROS [169].

Although these externally triggered treatments are still under development, they offer promising new strategies for implant sterilization. Intelligent antibacterial surfaces that activate only when required will be of significant interest [170]. The combined use of multiple antibacterial effects is becoming more common. However, it is important to note that while both photocatalytic and sonodynamic antibacterial effects generate ROS to kill bacteria, excessive ROS can be cytotoxic [171]. Given the complex nature of the human body, designing multi-functional TiO_2_ nano-pillar array surfaces that balance antibacterial effects with cytocompatibility and osteogenic activity is of strategic importance for medical implants [172].

While TiO_2_ nanomaterials have been extensively studied for their photocatalytic antibacterial effects, concerns remain regarding their biotoxicity and long-term stability. Studies have indicated that TiO_2_ nanomaterials possess low biotoxicity when introduced into the body, with safety largely dependent on their size and crystal form [46]. Pharmacokinetic studies have revealed that intravenous administration of TiO_2_ NPs does not adversely affect the health of mice, although these particles can accumulate in certain organs [46,173,174]. Additionally, the mechanical stability of TiO_2_ micro/nano-structured surfaces remains a critical issue that has not been thoroughly investigated. Long-term stability of implants necessitates robust mechanical properties [175], as any detachment of nanostructures during implant use may trigger cytotoxic reactions [31]. Therefore, considering long-term mechanical stress and wear across various application scenarios is crucial for implant applications [175,176].

Conversely, the osseointegration capacity of an implant significantly influences its long-term success within the body [160]. It is well documented that micro/nano-structured surfaces often exhibit superior osseointegration and bone regeneration capabilities [31,160,177]. For instance, TiO_2_ nano-pillar arrays enhance cell adhesion and promote osteogenic differentiation [32,129,133]. The morphological parameters of TiO_2_ nanostructures crucially affect cell attachment and proliferation [128]. Although research on TiO_2_ biomimetic micro/nano-structured surfaces is still at the preclinical stage, it represents an essential future direction in the field of nanoengineered implants [31,160]. Despite numerous challenges, such as parameter optimization, preclinical trials, and clinical trials, before the cost-effective mass production of TiO_2_ micro/nano-structures, any proactive step toward bridging the gap between research and practical application is valuable.

## 7. Conclusions

Renowned for their exceptional properties, Ti and its alloys are widely used on medical implants. However, implant-associated bacterial infections present significant challenges to patient recovery and public health. Additionally, the overuse of antibiotics has intensified the difficulty of treating implant-related infections. The formation of bacterial biofilms and the rise of drug-resistant bacteria have led to a crisis in traditional antibiotic treatments, making the development of non-antibiotic antibacterial strategies increasingly urgent.

Nature-inspired micro/nano-structured surfaces offer a unique mechanical antibacterial mechanism, presenting a promising approach that does not contribute to antibiotic resistance. The physical-mechanical interactions between micro/nano-structured surfaces and bacteria not only eliminate bacteria but also prevent bacterial adhesion and biofilm formation. Incorporating micro/nano-structures onto Ti surfaces can endow implants with inherent antibacterial properties. However, achieving highly effective antibacterial Ti implants solely through micro/nano-structured surfaces remains challenging.

The remarkable photocatalytic properties of TiO_2_ have been harnessed successfully in the antibacterial field. Moreover, TiO_2_ enhances the biocompatibility of Ti and offers unparalleled advantages for implant applications, making TiO_2_ micro/nano-structured surfaces highly valuable in implant research and applications. TiO_2_ exists in various crystal structures, each with distinct properties. Anatase demonstrates superior photocatalytic activity, while rutile exhibits lower cytotoxicity. Under specific light stimulation, TiO_2_ can generate ROS for antibacterial purposes, representing a non-antibiotic antibacterial approach. Thus, TiO_2_ micro/nano-structured surfaces, which combine photocatalytic activity with passive mechanical bactericidal effect, warrant further exploration.

Hydrothermal method is a highly effective method for preparing TiO_2_ micro/nano-structured surfaces. Both alkaline and acidic hydrothermal methods produce TiO_2_ micro/nano-structures with varied topographies. Alkaline hydrothermal method produces various micro/nano-structured surface topographies, while acidic hydrothermal method offers versatility across different substrates. By adjusting the parameters of the hydrothermal process, TiO_2_ micro/nano-structured surfaces with distinct topographies can be achieved. Understanding the impact of these preparation parameters on topography is crucial for developing efficient antibacterial TiO_2_ micro/nano-structured surfaces.

This review highlights recent advancements in hydrothermal method for the synthesis of TiO_2_ micro/nano-structures and emphasizes the antibacterial potential and future prospects of TiO_2_ nano-pillar array surfaces for Ti implants. These TiO_2_ nano-pillar arrays offer passive and sustained mechanical antibacterial properties. Additionally, their antibacterial efficacy can be further enhanced by integrating photocatalytic strategies. TiO_2_ micro/nano-structured surfaces with combined antibacterial capabilities are highly popular in medical applications.

In-depth investigations into the bactericidal mechanisms and osteogenic properties of TiO_2_ nano-pillar arrays are crucial for understanding surface–bacteria and surface–cell interactions, enabling the systematic design of more effective interfaces. Future research will focus on modifying TiO_2_ micro/nano-structured surfaces, with particular attention on emerging physical stimulated antibacterial effects. These potential advancements could lead to substantial progress in the surface modification of Ti implants. TiO_2_ micro/nano-structured surfaces with versatile antibacterial properties represent a promising avenue for the future design of implant surfaces.

## Figures and Tables

**Table 1:** The advantages and drawbacks of different biofabrication methods for preparing TiO_2_ micro/nano-structures.

Methods	Advantages	Drawbacks	Topographies	Ref.
EAO	1. Simple reaction process 2. Economically feasible 3. Controllable topography	Toxic reaction solvent	TiO_2_ nano-tubes TiO_2_ porous surfaces	[115,123]
CVD	1. Simple equipment 2. Diversified precursors 3. Highly uniform single crystal	1. Strict experimental conditions 2. High cost	TiO_2_ nano-pillars TiO_2_ nano-wires	[114,117]
Template Method	1. Controllable topography 2. Diverse template materials	1. Many experimental steps 2. Low productivity	TiO_2_ nano-tubes TiO_2_ nano-pillars	[88,118]
Sol-gel Method	1. Low processing temperature 2. Easy control of morphology 3. High purity	1. Long process duration 2. High cost of raw materials	TiO_2_ NPs	[113,119]
Laser Ablation	1. High crystalline quality 2. Suitable for extensive materials	1. Massive price 2. Low yield	TiO_2_ NPs	[122,125]
Electrostatic Spinning	Scalable production	1. Polycrystalline characteristics 2. High resistance 3. Poor surface adhesion	TiO_2_ nano-fiber surfaces	[114,120]
Hydrothermal Method	1. Simplicity 2. Low cost 3. High conversion rate 4. Environmentally friendly	1. Slow reaction kinetics 2. Long reaction duration	TiO_2_ nano-pillar arrays TiO_2_ nano-wire arrays	[124,126]

**EAO**: This method is characterized by a short preparation duration and results in relatively uniform sample topography, primarily used to fabricate TiO_2_ nano-tubes and TiO_2_ porous surfaces [115,123]. **CVD**: This method produces highly uniform single-crystal TiO_2_ nano-pillars and nano-wires of high quality [114,117]. However, the process is costly and equipment-intensive, limiting its large-scale production [117]. **Template Method**: This approach involves creating templates through other methods, synthesizing TiO_2_ nano-tubes, nano-pillars, or other structures within these templates, and then removing the templates via chemical etching [88,118]. It requires more experimental steps, has lower yields, and the resulting product topography is mainly determined by the template [118]. **Sol-gel Method**: Often used for synthesizing TiO_2_ NPs, this method can be combined with the template method to create TiO_2_ nano-wire surfaces [113,119]. **Laser Ablation:** This method uses laser beam ablation to remove material from a solid surface [122]. Traditional laser ablation presents several challenges, making liquid laser ablation a simple and versatile approach for synthesizing various NPs [122,125]. **Electrostatic Spinning**: Commonly used to produce TiO_2_ nano-fiber surfaces, this method is simple and suitable for large-scale production [114,120]. However, the resulting TiO_2_ is usually polycrystalline, and the nano-fibers may not adhere well to the surface [120]. **Hydrothermal Method**: Compared to other techniques, hydrothermal method has gained prominence due to its simplicity, low cost, and high yield [124]. The hydrothermal method is environmentally friendly and can convert amorphous TiO_2_ into single-crystal TiO_2_ [126]. It facilitates the preparation of TiO_2_ micro/nano-structured surfaces in various forms.

**Table 2:** TiO_2_ micro/nano-structured surfaces with different crystal structures and topographies prepared by alkaline and acidic hydrothermal methods. NaOH: Sodium Hydroxide. CMTFs: Chiral Mesostructured Films. NP: Nanoparticle. TMAOH: Tetramethylammonium Hydroxide. HCl: Hydrochloric Acid.

Methods	Reactive Solutions	Ti Sources	Crystal Structures	Topographies	Ref.
Alkaline Hydrothermal Method	NaOH (7–10 M)	Ti Plate	Anatase	Nano-tube Films	Luo 2014 [130]
	NaOH (10 M)	Ti Foil	Rutile	Nano-tube Films	Sun 2021 [131]
	NaOH (0.1 M)	Ti Foil	Anatase	CMTFs	Zhang 2020 [132]
	NaOH (10 M)	TiO_2_ NP Compacts	Anatase	Nano-wire Arrays	Zhang 2022 [128]
	NaOH (1–2 M)	Ti Plate	Anatase & Rutile	Nano-wire Arrays	Jaggessar 2018 [133]
	NaOH (1 M)	Ti Disk	/	Nano-spikes	Gao 2020 [51]
	NaOH (1 M)	Ti Plate	/	Nano-spikes	Huo 2023 [52]
	NaOH (0.5–4 M)	Ti Plate	Anatase	Nano-sheet Arrays	Li 2022 [53]
	NaOH (0.5–4 M)	Ti Plate	Anatase	Nano-belt Arrays	Li 2022 [53]
	NaOH (0.5–4 M)	Ti Plate	Anatase	Nano-wire Film	Li 2022 [53]
	TMAOH (1 M)	Ti Plate	Anatase	Nano-shovel Arrays	Dong 2010 [134]
	TMAOH (1 M)	Ti Plate	Anatase	Nano-shovel Arrays	Zhang 2022 [135]
	NaOH (1 M)	Ti Foil	Anatase	Nano-pillar Arrays	Zhang 2021 [129]
	NaOH (1 M)	Ti Plate	Anatase	Nano-pillar Arrays	Zhang 2021 [136]
	NaOH (1 M)	Ti Foil	Anatase	Nano-pillar Arrays	He 2022 [137]
Acidic Hydrothermal Method	HCl (4 M)	TiCl_4_	Rutile	Nano-pillar Arrays	Khizir 2021 [138]
	HCl (4 M)	Ti (IV) Butoxide	Rutile & Anatase	Nano-pillar Arrays	Prathan 2020 [127]
	HCl (6 M)	Ti Isopropoxide	Rutile	Nano-pillar Arrays	Ben 2021 [139]
	HCl (6 M)	Ti (IV) Butoxide	Rutile	Nano-pillar Arrays	Meshesha 2022 [140]
	HCl (6 M)	Ti (IV) Butoxide	Rutile	Nano-wire Arrays	Meshesha 2022 [140]
	HCl (7.5 M)	Tetrabutyl Titanate	Rutile	Nano-pillar Arrays	Wang 2022 [141]
	HCl (0.5 M)	Ti Foil	Rutile & Anatase	Nano-pillar Arrays	Xing 2022 [142]

## Data Availability

The original contributions presented in the study are included in the article, further inquiries can be directed to the corresponding authors.
